# Phase I study of sapanisertib with carboplatin and paclitaxel in mTOR pathway altered solid malignancies

**DOI:** 10.1038/s41698-023-00369-w

**Published:** 2023-04-18

**Authors:** Omar Alhalabi, Roman Groisberg, Ralph Zinner, Andrew W. Hahn, Aung Naing, Shizhen Zhang, Apostolia M. Tsimberidou, Jordi Rodon, Siqing Fu, Timothy A. Yap, David S. Hong, Ming Sun, Yunfang Jiang, Shubham Pant, Amishi Y. Shah, Amado Zurita, Nizar M. Tannir, Raghunandan Vikram, Jason Roszik, Funda Meric-Bernstam, Vivek Subbiah

**Affiliations:** 1 Department of Genitourinary Medical Oncology, Division of Cancer Medicine, University of Texas MD Anderson Cancer Center, Houston, TX USA; 2 Department of Investigational Cancer Therapeutics, Division of Cancer Medicine, University of Texas MD Anderson Cancer Center, Houston, TX USA; 3 Department of Medical Oncology, Rutgers University, New Jersey, NJ USA; 4 Department of Thoracic Oncology, University of Kentucky, Lexington, KY USA; 5 Division of Cancer Medicine, University of Texas MD Anderson Cancer Center, Houston, TX USA; 6 Department of Abdominal Imaging, Division of Diagnostic Imaging, The University of Texas MD Anderson Cancer Center, Houston, TX USA; 7 Department of Genomic Medicine, Division of Cancer Medicine, University of Texas MD Anderson Cancer Center, Houston, TX USA; 8 Department of Melanoma Medical Oncology, Division of Cancer Medicine, University of Texas MD Anderson Cancer Center, Houston, TX USA

**Keywords:** Drug development, Drug safety

## Abstract

Pre-clinically, the mTORC1/2 inhibitor sapanisertib restored sensitivity to platinums and enhanced paclitaxel-induced cancer cell killing. NCT03430882 enrolled patients with mTOR pathway aberrant tumors to receive sapanisertib, carboplatin and paclitaxel. Primary objective was safety and secondary objectives were clinical response and survival. One patient had a dose-limiting toxicity at dose level 4. There were no unanticipated toxicities. Grade 3–4 treatment-related adverse events included anemia (21%), neutropenia (21%), thrombocytopenia (10.5%), and transaminitis (5%). Of 17 patients evaluable for response, 2 and 11 patients achieved partial response and stable disease, respectively. Responders included a patient with unclassified renal cell carcinoma harboring EWSR1-POU5F1 fusion and a patient with castrate resistant prostate cancer harboring PTEN loss. Median progression free survival was 3.84 months. Sapanisertib in combination with carboplatin plus paclitaxel demonstrated a manageable safety profile, with preliminary antitumor activity observed in advanced malignancies harboring mTOR pathway alterations.

## Introduction

The aberrant activation of the phosphoinositide 3-kinase/protein kinase B/mammalian target of rapamycin (PI3K/AKT/mTOR) signaling pathway is associated with malignant transformation and resistance to apoptosis^1^. Hence, mTOR, which is the catalytic protein that nucleates both the mTOR complex 1 (mTORC1) and mTOR complex 2 (mTORC2), has become a target of therapy development. The US Food and Drug Administration has approved the two rapamycin analogues (“rapalogs”), temsiromlimus and everolimus for the treatment of multiple solid cancers including breast cancer and renal cell carcinoma (RCC)^2–5^. Rapalogs, however, serve as allosteric inhibitors of mTOR within the mTORC1 complex only and lack of mTORC2 inhibition, which has been proposed as a mechanism of resistance^6^. Therefore, a new generation of dual mTORC1/2 inhibitors has been developed with promising activity in preclinical models including those with acquired rapamycin resistance^7–9^. Sapanisertib (CB-228,TAK-228, MLN0128) is an orally bioavailable potent inhibitor of both mTORC1 and mTORC2 that demonstrated a manageable safety profile, with preliminary antitumor activity observed in renal cell carcinoma (RCC) and endometrial cancer^10^.

Anti-angiogenic effects of mTOR inhibition were shown to synergize with the antitumor and antiangiogenic effects of weekly paclitaxel^11^. However, this synergy was observed with sequential, rather than simultaneous, mTOR blockade after chemotherapy^11^. In vivo modeling confirmed that the combination of paclitaxel on Day 1 and sapanisertib given on Days 2–4 resulted in improved tumor growth inhibition compared to concomitant administration of the two agents^12^. Furthermore, platinum resistance has been shown to be related to activating phosphorylation of AKT and preclinical data demonstrated that mTORC1/2 inhibition restores sensitivity to platinum chemotherapy^13^. Taken together, preclinical data support the sequential addition of sapanisertib to carboplatin plus paclitaxel. Clinical tolerability for different schedules of the combination is expected to be different from single agent sapanisertib. Here, we evaluate a range of dosing schedules in this phase 1 study (NCT03430882) of sapanisertib in combination with carboplatin plus paclitaxel in patients with advanced solid malignancies.

## Results

### Characteristics of patients

From May 2018 to March 2020, 19 patients with advanced or metastatic solid malignancies that were refractory to standard-of-care therapy were enrolled. Demographics and disease characteristics are summarized in Table 1. The median age was 59 (range 33–74) and 58% were male. At baseline, about a quarter of patients (26%) had more than 3 metastatic sites of disease. Breast adenocarcinoma was the most represented primary disease site (32%), the majority of which were hormone receptor positive and HER2/neu negative (5 of 6 patients), followed by lung carcinoma (16%) and sarcoma (16%). All patients had ECOG performance score of 1 and the majority (63%) had three or more prior lines of therapy.

**Table 1:** Patient baseline characteristics and demographics.

Characteristics	(*N* = 19)
*Age*	
Median	59
Range	33–74
*Sex (%)*	
Male	11 (58)
Female	8 (42)
*Ethnicity (%)*	
White	18 (95)
Asian	1 (5)
*Number of metastatic sites (%)*	
≤3	14 (74)
>3	5 (26)
*Primary disease site (%)*	
Breast adenocarcinoma^a^	6 (32)
Lung carcinoma^b^	3 (16)
Sarcoma^c^	3 (16)
Renal cell carcinoma	1 (5)
Head and neck squamous cell carcinoma	1 (5)
Others^d^	5 (26)
*ECOG PS (%)*	
0	0 (0)
1	19 (100)
*Number of prior therapies (%)*	
1–2	7 (37)
3–9	12 (63)

*ECOG PS* Eastern Cooperative Oncology Group performance status.

^a^Breast adenocarcinoma receptor status was hormone positive, HER2 negative in 5 patients and triple negative in 1 patient.

^b^Lung carcinoma histology was non-small cell in 2 patients and carcinoid in 1 patient.

^c^Sarcoma types included a patient with synovial sarcoma, a patient with leiomyosarcoma, a patient with endometrial sarcoma.

^d^Other primary disease sites included patients with esophageal adenocarcinoma, urothelial cancer, plexiform fibrohistiocytic tumor of skin, prostate adenocarcinoma, and squamous cell carcinoma of anal margin, respectively.

### DLTs and MTD determination

Of the 19 patients treated, 4 patients were treated in dose level 1 (Carboplatin AUC 5 mg/mL•min every 3 weeks, Paclitaxel 40 mg/m^2^ weekly, TAK-228 2 mg Day 2–4, 9–11, 16–18). All 4 patients treated in dose Level 1 were DLT-evaluable with no DLT observed. Four patients were treated in dose level 2 (Carboplatin AUC 5 mg/mL min every 3weeks, Paclitaxel 40 mg/m^2^ weekly, TAK-228 3 mg Day 2–4, 9–11, 16–18). One patient was not DLT-evaluable, 3 patients were DLT-evaluable and no DLT was observed. Four patients were treated in dose level 3 (Carboplatin AUC 5 every 3 weeks, Paclitaxel 40 mg/m^2^, weekly, TAK-228 4 mg Day 2–4, 9–11, 16–18). All 4 patients were DLT evaluable and no DLT observed. Seven patients were treated in dose level 4 (Carboplatin AUC 5 mg/mL min every 3 weeks, Paclitaxel 60 mg/m^2^, weekly, TAK-228 4 mg Day 2–4, 9–11, 16–18). Among these patients treated in dose level 4, 1 patient was not evaluable for DLT, 1 experienced DLT (grade 4 thrombocytopenia lasting for more than a week) and 5 experienced no DLT.

### Treatment exposure and safety

There were no unanticipated safety issues associated with the use of study drugs. Grade 3–4 treatment-related AEs (TRAE) included anemia (4/19; 21%), neutropenia (4/19; 21%), thrombocytopenia (2/19; 10.5%), and transaminitis (1/19; 5%). There were no grade 5 AEs. Treatment-related AEs across study are summarized in Table 2. Grade 3–4 TRAEs were seen at dose levels 2, 3 and 4 (Table 3). Treatment-related serious adverse events (SAEs) were seen in one patient under dose level 2. AEs leading to discontinuation of trial therapy occurred in 3 (16%) patients. AEs requiring a dose interruption and dose reduction occurred in 11 (58%) and 7 (37%) patients, respectively.

**Table 2:** Treatment-related adverse events.

AE terms	Related to TAK-228	G1	G2	G3	G4	Related to Carbo/Taxol
ALT increased	POR	1	1	1		POR
Anorexia	POR	4				POR
AST increased	POR	1	1	1		POR
Cough	POR	1				
Diarrhea	POR	2				POR
Dry mouth	POR	1				
Dyspnea (SOB)	POR	2				
Fatigue	POR		4			POR
headache	POR	1				POR
Hyperglycemia	POR	3	2			
Hypertriglyceridemia	POR	4	1			
Increased HbA1C	POR	3				
Itching	POR	1				
Mouth Sore	POR	4				
Nausea	POR	11	4			POR
pneumonia	POR	1				
Rash	POR	5				
Stroke	POR	1				POR
Taste change	POR	1				
Vomiting	POR	6	1			POR
Weight loss	POR	4	1			POR
Anemia	Unlikely	5	10	4		POR
dehydration	Unlikely	1				POR
Facial Flushing	Unlikely	3				POR
Hair loss	Unlikely	1				POR
Hard of hearing	Unlikely	1				POR
Hiccups	Unlikely	1				POR
Neuropathy	Unlikely	2	1			POR
Neutrophil count decreased	Unlikely	4	8	4	2	POR
Platelet Count decreased	Unlikely	8	5	2	1	POR
White blood Count decreased	Unlikely	8	8	4		POR

*POR* possibly related, *G* grade.

**Table 3:** Overall safety profile.

	Dose escalation			
	Dose level 1	Dose level 2	Dose level 3	Dose level 4
	*n* = 4	*n* = 4	*n* = 4	*n* = 7
	Paclitaxel 40 mg/m^2^, Carboplatin 5AUC, TAK-228 2 mg	Paclitaxel 40 mg/m^2^, Carboplatin 5AUC, TAK-228 3 mg	Paclitaxel 40 mg/m^2^, Carboplatin 5AUC, TAK-228 4 mg	Paclitaxel 60 mg/m^2^, Carboplatin 5AUC, TAK-228 4 mg
Any AE, *n* (%)	4 (100)	4 (100)	4 (100)	7 (100)
Any grade ≥ 3 AE, *n* (%)	0 (0)	4 (100)	3 (75)	3 (43)
Treatment-related AE, *n* (%)	4 (100)	4 (100)	3 (75)	7 (100)
Treatment-related grade ≥3 AE, *n* (%)	0	3 (75)	1 (25)	3 (43)
SAE, *n* (%)	0	3 (75)	2 (50)	2 (29)
Treatment-related SAEs, *n* (%)	0	1 (25)	0	0
AEs resulting in discontinuation, *n* (%)	1 (25)	0	1 (25)	1 (14)
AEs resulting in dose reduction, *n* (%)	0	0	4 (100)	3 (43)
AEs resulting in dose interruption, *n* (%)	1 (25)	3 (75)	3 (75)	4 (57)
On-study deaths, *n* (%)	0	0	0	0

*AE* adverse event, *SAE* serious adverse event.

### Antitumor activity

**Fig. 1:**
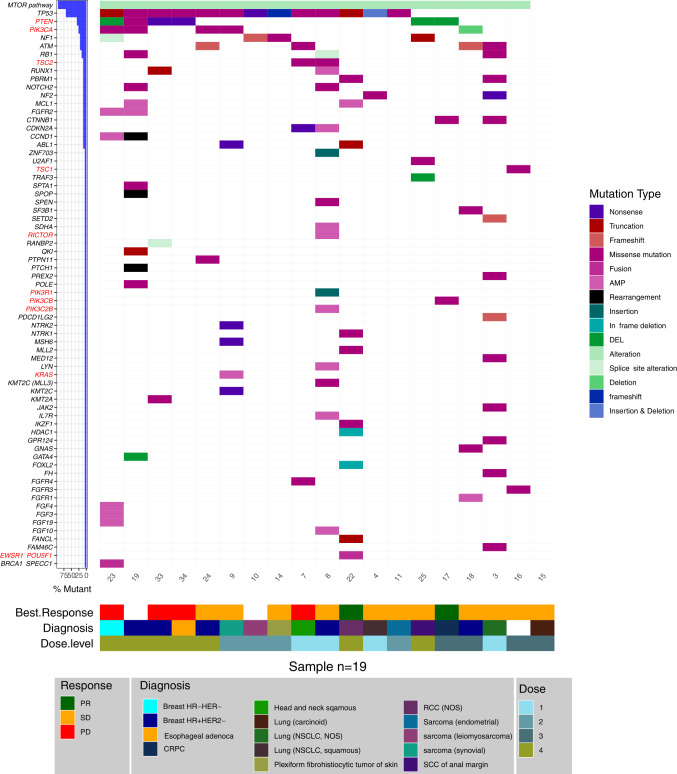
Oncoplot representing diagnosis, best response, dose level and targeted sequencing of DNA alterations and RNA fusion products in enrolled patients. Highlighted in red are genes implicated in the mTOR pathway. Key: PR partial response, SD stable disease, PD progressive disease, HR hormone receptor, HER2 human epidermal growth factor receptor 2, CRPC castration resistant prostate cancer, NSCLC non-small cell lung cancer, RCC renal cell cancer, SCC squamous cell carcinoma.

At data cut-off (Feb 5, 2021), all patients are no longer receiving study treatment. Median PFS was 3.84 months. Tumor responses across study participants in correlation with molecular profile, dose level and histology are summarized in Fig. 1. Of the 19 patients treated, 11 patients’ best overall response (BOR) was stable disease (SD); 2 patients’ BOR was partial response (PR) in RCC (37% decrease of target lesions) and in prostate cancer (34% decrease of target lesions), and 4 patients’ BOR was progressive disease. Disease control rate occurred in 13 (68%) patients. 2 patients were not evaluable for response due to withdrawal of consent prior to restaging.

### Clinical response in a patient with unclassified RCC harboring *EWSR1-POU5F1* fusion t(6;22) (p21;q12)

**Fig. 2:**
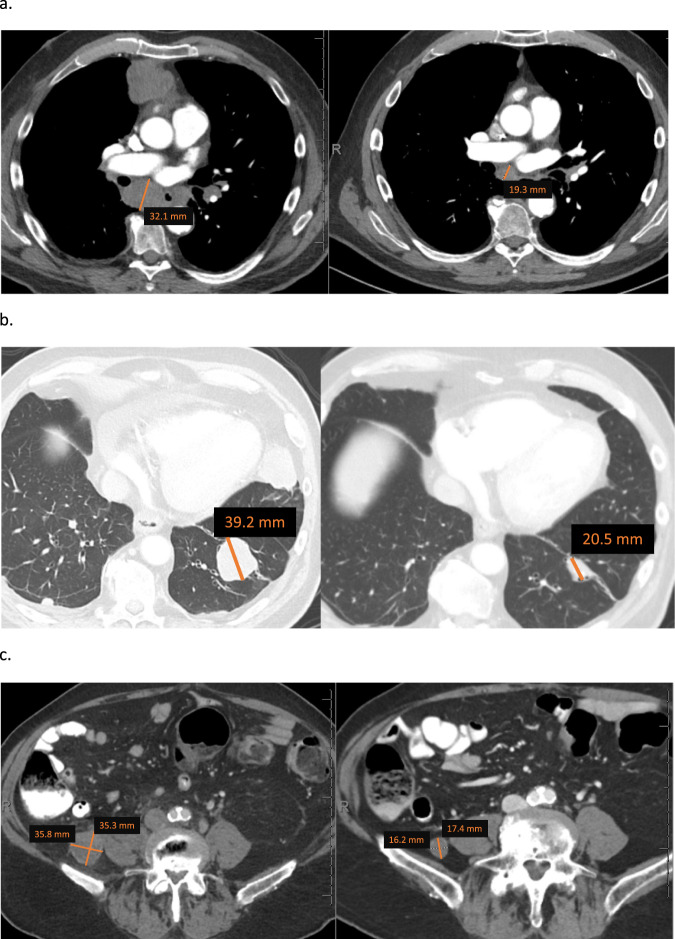
Representative sections of computerized tomography in patient #22 with unclassified renal cell carcinoma harboring EWSR1-POU5F1 fusion t(6;22)(p21;q12). Panels (**a**), (**b**), (**c**) show partial response per RECIST version 1.1 in mediastinal adenopathy (**a**), lung metastasis (**b**) and pelvic adenopathy (**c**) in Left and right panels reflect baseline and nadir after therapy, respectively.

Here, we describe a patient (#22) who presented with metastatic unclassified RCC to bulky abdominal lymph nodes and numerous nodules in lung bases (Fig. 2). A lung nodule biopsy revealed poorly differentiated carcinoma. Patient underwent diagnostic upfront nephrectomy which showed 12 cm poorly differentiated carcinoma invasive into renal sinus, perinephric adipose tissue, and renal vein. The tumor showed positive staining for CD56, PAX-8, and CAM5.2. Diagnosis was made as unclassified RCC. Molecular profiling revealed an *EWSR1-POU5F1* fusion. The patient received frontline nivolumab plus ipilimumab with lack of response. Upon treatment with trial regimen, tumors demonstrated a dramatic, deep PR within 5 cycles of a trial regimen (Fig. 2). Patient remained on therapy for more than 20 months then discontinued therapy due to declining performance status. Mutations in *FANCL, PBRM1, NTRK1, MLL2, IKZF1, HDAC1*, and *FOXL2* were among the unique mutations found in this patient and not found in non-responder patients (Fig. 1).

### Clinical response in a patient with metastatic castrate-resistant prostate cancer harboring *PTEN* loss

**Fig. 3:**
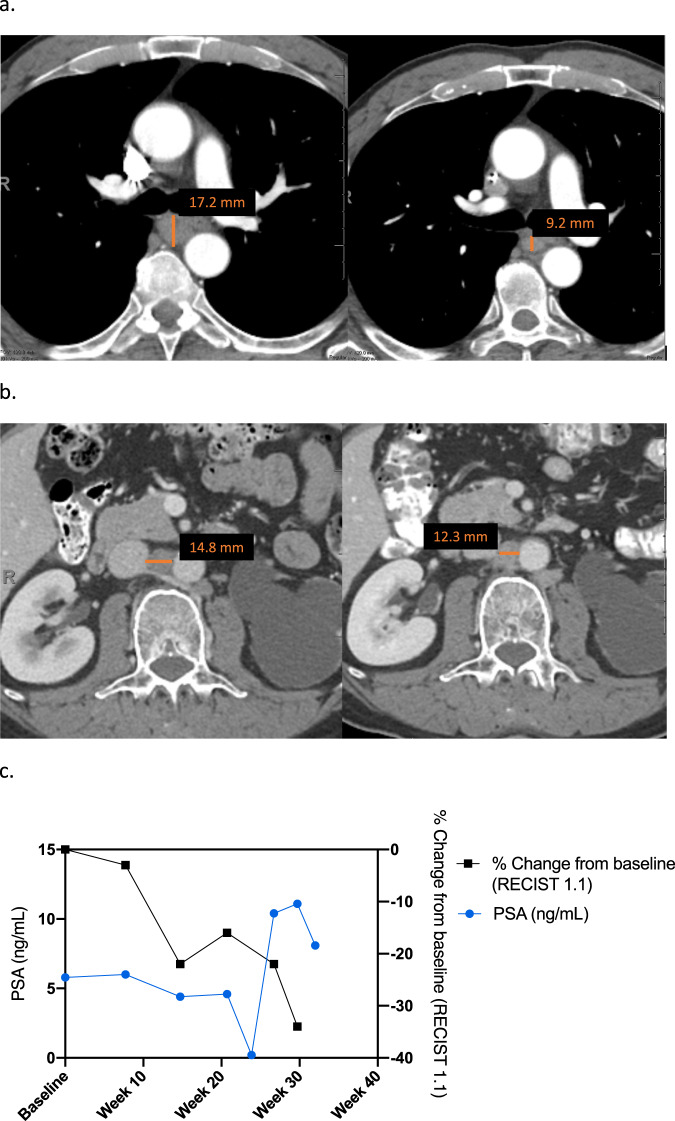
Representative sections of computerized tomography and PSA trend in patient #17 with metastatic castrate resistant prostate cancer harboring PTEN deletion. Panels (**a**), (**b**) show response in mediastinal (**a**) and retroperitoneal (**b**) nodal metastases. **c** XY plot of the PSA (ng/mL) levels (blue, left *Y* axis) and % change from baseline (RECIST v1.1) in relation to time. Plot was created using GraphPad Prism version 9.2.0 for Mac OS, GraphPad Software, San Diego, California, USA, www.graphpad.com.

Furthermore, we describe a patient (#17) who had developed castrate-resistant prostate cancer (mCRPC) metastatic to mediastinal and abdominal lymph nodes. He had received prior enzalutamide, abiraterone, and cabazitaxel. Molecular profiling revealed *PTEN* loss, which triggered the interest in mTOR targeting therapy. Upon treatment with trial regimen, tumors demonstrated PR per RECIST v1.1 and PSA50 criteria (Fig. 3). Time to treatment failure was 7 months. A mutation in *PIK3CB* was among the unique mutations found in this patient and not found in non-responder patients (Fig. 1).

## Discussion

In this open-label, Phase 1 study, the safety profile of triplet sapanisertib plus carboplatin and paclitaxel was characterized and shown to be manageable and consistent with the toxicity anticipated of either sapanisertib or carboplatin plus paclitaxel. Sapanisertib showed preliminary antitumour activity in a patient with unclassified RCC harboring an *EWSR1-POU5F1* fusion and a patient with prostate adenocarcinoma harboring a *PTEN* deletion. The triplet MTD was determined as carboplatin AUC 5 mg/mL•min every 3 weeks, Paclitaxel 60 mg/m^2^, weekly, and TAK-228 4 mg Day 2–4, 9–11, 16–18. Given that majority of patients in our study were white, 4 mg dose seems consistent with a prior study showing RP2D of sapanisertib in East Asian patients (3 mg QD) was lower than in Western patients (4 mg QD)^14^. The safety profile of sapanisertib in this phase 1 study was generally manageable across all schedules, and tolerability was greater with increased intermittence of dosing. Common AEs related to sapanisertib across all schedules in both phases included hyperglycemia, nausea, and vomiting, which are all well-known side effect of PI3K/mTOR pathway inhibition^15^.

**Fig. 4:**
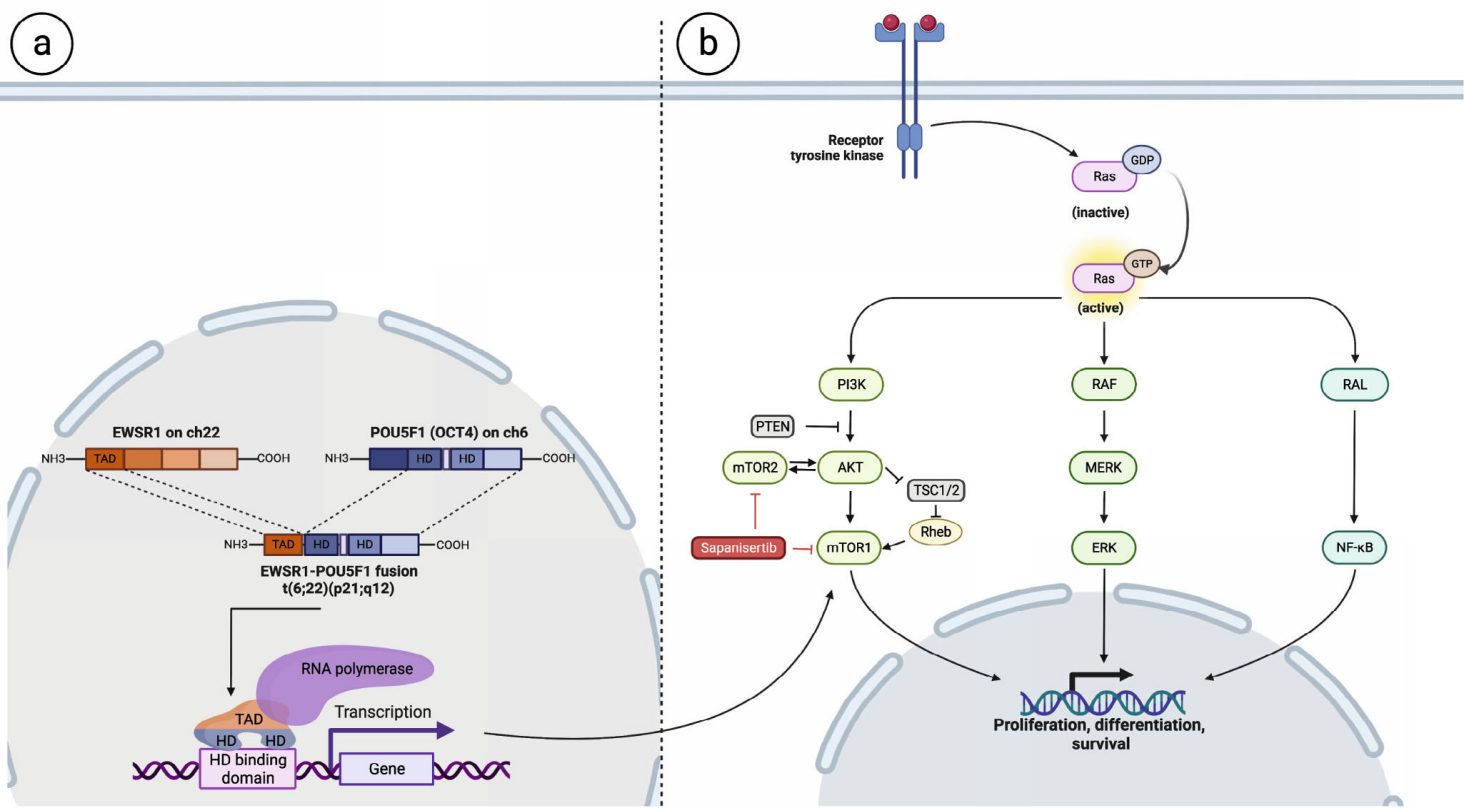
Hypothesized interaction between genomic alteration and sapanisertib in the two responders. **a** Schema highlighting the EWSR1-POU5F1 fusion t(6;22)(p21;q12) peptide. POU5F1 (OCT4) belongs to the homeobox gene superfamily and encodes a transcription factor that act as a master regulator of development. POU5F1 has preserved homaeodomains (HD) that bind DNA. The fusion peptide brings the HD of POU5F1 in proximity to N-terminal transcriptional activation domain (TAD) of EWSR1. This fusion peptide hypothetically leads to enrichment of EWSR1-related signaling pathways including the mTOR signaling pathway, p53 signaling pathway, and WNT signaling pathway. **b** Schema depicting the downstream signaling pathways following the activation of receptor tyrosine kinase including the RAF/MERK/ERK and the PI3K/AKT/mTOR pathways. Figure was created using biorender.com.

*EWSR1* fusions are pathognomonic features of Ewing sarcoma (most commonly with Fli-1 proto-oncogene, ETS transcription factor [*FLI1*] and occasionally with ETS transcription factor ERG [ERG])^16^. On the other hand, *POU5F1* (*OCT4*) belongs to the homeobox gene superfamily and encodes a transcription factor that act as a master regulator of development, especially during embryogenesis^17^. *POU5F1* has preserved homeodomains (HD) that bind DNA. The fusion peptide brings the HD of *POU5F1* in proximity to N-terminal transcriptional activation domain (TAD) of *EWSR1*. This fusion peptide hypothetically (Fig. 4) leads to enrichment of *EWSR1*-related signaling pathways including the mTOR signaling pathway, p53 signaling pathway, and WNT signaling pathway^18^. Furthermore, *EWSR1* fusion tumors have been shown to have constitutive activation of the mTOR pathway and respond to mTOR inhibition^19,20^, perhaps explaining the response seen to sapanisertib in our patient. The molecular similarity between our unclassified RCC case harboring *EWSR1-POU5F1* fusion and Ewing sarcoma with *EWSR1-FLI1* fusion was the rationale for the platinum-based combination^21^ for our patient with mRCC.

Downstream signaling pathways following the activation of receptor tyrosine kinase include the activation of the well-known RAF/MERK/ERK and the PI3K/AKT/mTOR pathways which is controlled in part by the tumor-suppressor gene: *PTEN*. *PTEN*-deficient tumors have been reported to have enhanced sensitivity to the inhibition of mTOR pathways^22^. The hypothesized (Fig. 4) downstream activation of mTOR signaling in our patient with mCRPC was the rationale for choosing this trial regimen for his therapy. In a murine model, mTOR complex 2 seems to be required for the development of prostate cancer induced by *Pten* loss^23^, which further supports the use of sapanisertib. In addition, the observed clinical benefit despite his heavily pretreated disease with cabazitaxel suggests the plausible role of sapanisertib in his response.

Our study was limited due to the lack of correlative data due to the nature of the study and biopsies not being mandated. Secondly, in the platinum-naïve PTEN-loss mCRPC patient, we were unable to confirm if the response seen to the combination therapy would have occurred with single agent sapanisertib therapy alone or the role of the concurrent PIK3CB alteration. A recent study showed significant co-occurrence between PTEN and PIK3CA/PIK3CB/PIK3R1 alterations in prostate cancer^24^. Further in vitro work is necessary to better isolate the impact of each of these mutations on the potential sensitization to mTORC1/2 inhibition. Our study was a proof-of-concept trial to show that a dual mTOR1/2 inhibitor could be safely combined with chemotherapy.

In conclusion, sapanisertib plus chemotherapy was generally well tolerated with no unexpected safety signals across the various schedules studied. The triplet MTD was determined as carboplatin AUC 5 mg/mL•min every 3 weeks, Paclitaxel 60 mg/m^2^, weekly, and TAK-228 4 mg Day 2–4, 9–11, 16–18. Preliminary antitumour activity was observed in two patients with unclassified mRCC and mCRPC, respectively. Although single-agent sapanisertib has been preliminarily reported to have modest activity in patients with previously treated, metastatic clear-cell RCC (NCT03097328), our findings will help better inform sapanisertib plus chemotherapy combination dosing strategies in future advanced solid tumor clinical trials.

## Methods

### Study design and treatment

**Fig. 5:**
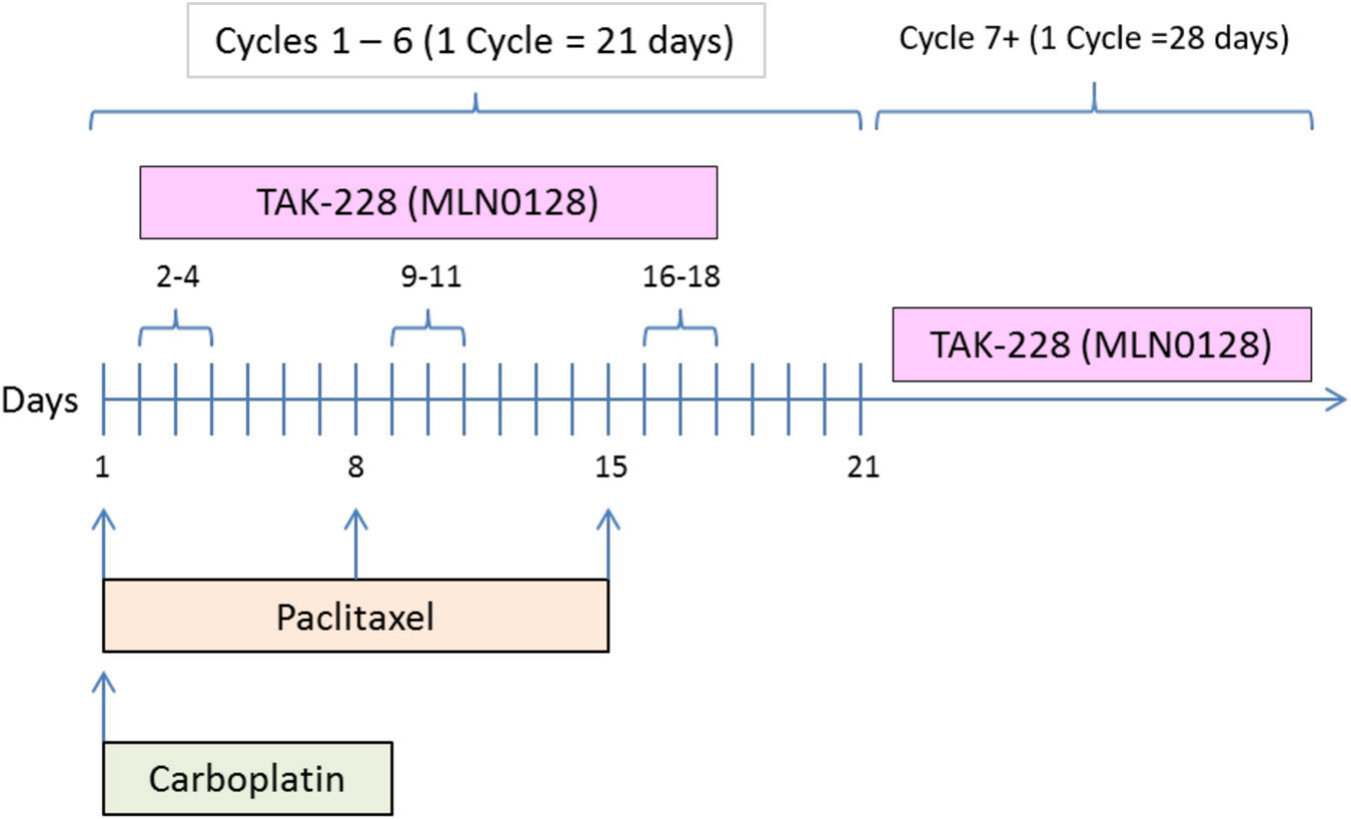
Trial schema (NCT03430882). Patients were treated with as many as six, 21-day cycles of the combination regimen. The regimen is comprised of carboplatin AUC 5 intravenously every 3 weeks on Day 1 of each cycle plus sapanisertib (TAK-228 or MLN0128) daily on Day 2–4 of each week at 2 mg, 3 mg and 4 mg orally and paclitaxel 40 mg/m2, 60 mg/m2, 80 mg/m2 QW IV on Day 1, 8, and 15 of each cycle. Evaluation of the composite toxicity/efficacy endpoint was done at Day 21 of treatment. After the 6 cycles were completed, patients were treated to progression with sapanisertib 3 mg PO daily.

This Phase 1, open-label, dose-escalation study aimed to determine the safety, tolerability and preliminary efficacy of sapanisertib in combination with carboplatin plus paclitaxel in patients with advanced solid malignancies. Patients were treated with as many as six, 21-day cycles of the combination regimen (Fig. 5). The regimen was comprised of carboplatin (targeting area under the curve -AUC- 5 mg/mL•min) intravenously every 3 weeks on Day 1 of each cycle plus sapanisertib (TAK-228 or MLN0128) daily on Day 2–4 of each week at 2 mg, 3 mg and 4 mg orally and paclitaxel 40 mg/m2, 60 mg/m2, 80 mg/m2 QW IV on Day 1, 8, and 15 of each cycle. After the 6 cycles were completed, patients were treated to progression with sapanisertib 3 mg PO daily. The rationale behind limiting chemotherapy to six cycles was to avoid overlapping cumulative toxicity. Sapanisertib was continued as a maintenance therapy given the well-known resistance emerging from chemotherapy. This protocol utilized a standard 3 + 3 design. Three patients were treated per dose level. DLT was defined if the events occur between Day 1 and Day 21 of the first cycle. If none of the patients experienced DLT, the next cohort of three patients were treated at the next higher dose level. If one of three patients treated at a dose experiences DLT, then that cohort was expanded to a total of six patients. If the incidence of DLT among those six patients is one in six, then the next cohort was treated at the next higher dose level. If two or more of six patients treated at a dose level experience DLT, then the Maximum Tolerated Dose (MTD) was considered to have been exceeded. Two or three more patients (for a total of 6) were treated at the next lower dose as described above unless six patients have already been treated at that dose. In summary, the MTD is defined as the highest dose studied in which the incidence of DLT was less than 33%. The patient must have completed at least 66% of planned doses of all drugs to be evaluable for a DLT.

A DLT was defined, as predetermined in the study protocol, with the following: any grade ≥3 non-hematologic toxicity (except inadequately treated grade 3 nausea and/or vomiting and grade 3 diarrhea [all patients should have received optimal antiemetic and/or antidiarrheal prophylaxis and/or treatment], grade 3 hyperglycemia lasting ≤14 days [all patients should have received optimal anti-glycemic treatment, including insulin) and grade 3 rash lasting ≤3 days [all patients should have received topical steroid treatment, oral antihistamines and pulse oral steroids, if necessary]); grade 4 neutropenia lasting >7 days in the absence of growth factor support; grade 4 neutropenia of any duration accompanied with fever ≥38.5 °C and/or systemic infection; grade 3 thrombocytopenia with bleeding; any other grade ≥4 hematologic toxicity.

In the event of any adverse event defined as DLT attributable to the study drug(s), the drug(s) was(were) withheld. If the event resolved to Grade ≤ 2 within 28 days of interrupting therapy, the patient could resume study treatment at the next lower dose level. If sapanisertib (and other study agent(s), if applicable) dosing was delayed for >28 consecutive days for treatment-related toxicity, despite supportive treatment per standard clinical practice, or more than 2 dose reductions were required in a patient, sapanisertib (and other study agent(s), if applicable) therapy was stopped, the patient was discontinued from the study and the follow-up visit was completed within 30 days of the last administration of sapanisertib (and other agent if applicable), whichever is discontinued last.

### Patients

Eligible patients were 18 years or older with solid tumor malignancy that is refractory to standard therapy, had an Eastern Cooperative Oncology Group (ECOG) performance status of 0 or 1, and had adequate bone marrow reserve, hepatic, renal and metabolic (fasting serum glucose ≤ 130 mg/dL and fasting triglycerides ≤ 300 mg/dL) function. Patients were required to have evaluable or measurable disease by Response Evaluation Criteria in Solid Tumors (RECIST) 1.1 criteria. Patients who have a history of brain metastases were eligible if their brain metastases had been treated (without evidence of progression or hemorrhage post-treatment), and if they had not taken dexamethasone 4 weeks prior to the first study drug administration and with no ongoing requirement for dexamethasone or antiepileptic drugs. Patients who had received carboplatin or paclitaxel within past 6 months, systemic corticosteroid therapy or proton pump inhibitors within past 7 days, dual PI3K/TORC1/2, mTORC1/2 inhibitors or mTORC1 inhibitors were not eligible. Additionally, patients with impaired cardiac function or significant active cardiovascular disease were also excluded.

### Assessments

The safety population was defined as all enrolled patients who received at least 1 dose of any study drug. The DLT-evaluable population was defined as all patients who either experience DLT during the DLT window or those who receive at least 66% of the planned study drug administrations in Cycle 1 and 2 and have sufficient follow-up data to allow the investigators to determine whether DLT occurred. The response-evaluable population was defined as all patients who had measurable disease according to RECIST version 1.1 at baseline who have received at least 66% of the planned doses of any study drug, and who have at least 1 available post-baseline response assessment per RECIST version 1.1. Response was assessed according to the RECIST v1.1^25^ after every two treatment cycles. Adverse events (AEs) were assessed using the National Cancer Institute Common Terminology Criteria for Adverse Events, v4.0. Patients were provided with a home blood glucometer to monitor their fasting blood glucose measurements to assess hyperglycemia as an on-target AE marker.

### Objectives and statistical plan

Primary study objectives are to determine the safety and tolerability of the combination of sapanisertib plus paclitaxel and carboplatin, and to determine the optimal dose triplet, or MTD, in patients with advanced cancers refractory to standard therapy. Secondary study objectives are clinical tumor response of this combination, and progression free survival (PFS). PFS was calculated from the start of trial therapy till progression or last follow up where imaging did not demonstrate progression. Statistical analyses are primarily descriptive and graphical in nature, with no formal statistical hypothesis testing.

### Molecular profiling

The molecular testing was part of clinical next generation sequencing (NGS) assays performed in the course of clinical care of the patient to identify targeted therapies for molecular matching. The biopsies were from sites that were considered safe by the interventional radiology. Samples were both from primary and metastatic sites. Biopsies were performed prior to trial therapy. NGS data were collected from five panels: FoundationOne (Foundation Medicine, Cambridge, MA) (*N* = 3), FoundationOne CDx (Foundation Medicine, Cambridge, MA) (*N* = 2), FoundationOne Liquid CDx (Foundation Medicine, Cambridge, MA) (*N* = 1), Guardant360 (Guardant Health, Palo Alto, CA) (*N* = 3), Liquid Biopsy Panel V1 (MD Anderson Cancer Center, Houston, TX) (*N* = 3), STGA-DNA 2018 (MD Anderson Cancer Center, Houston, TX) (*N* = 10), and Tempus xT (Tempus, Chicago, IL) (*N* = 2). A few patients had undergone multiple panel testing. The five panels included the following numbers of genes: FoundationOne (416), FoundationOne CDx (324), FoundationOne Liquid CDx (311), Guardant360 (74), Liquid Biopsy Panel V1 (70), STGA-DNA (147), and Tempus xT (648).

## Data Availability

The datasets supporting the results reported in this article, will be made available upon reasonable request from the corresponding author to researchers who provide a methodologically sound proposal. The data will be provided after its de-identification, in compliance with applicable privacy laws, data protection and requirements for consent and anonymization.

## References

[1] Vivanco I, Sawyers CL (2002). The phosphatidylinositol 3-Kinase AKT pathway in human cancer. Nat. Rev. Cancer.

[2] Baselga J (2011). Everolimus in postmenopausal hormone-receptor–positive advanced breast cancer. N. Engl. J. Med..

[3] Yao JC (2016). Everolimus for the treatment of advanced, non-functional neuroendocrine tumours of the lung or gastrointestinal tract (RADIANT-4): a randomised, placebo-controlled, phase 3 study. Lancet.

[4] Motzer RJ (2008). Efficacy of everolimus in advanced renal cell carcinoma: a double-blind, randomised, placebo-controlled phase III trial. Lancet.

[5] Hudes G (2007). Temsirolimus, Interferon Alfa, or both for advanced renal-cell carcinoma. N. Engl. J. Med..

[6] O’Reilly KE (2006). mTOR inhibition induces upstream receptor tyrosine kinase signaling and activates Akt. Cancer Res..

[7] Zheng B (2015). Pre-clinical evaluation of AZD-2014, a novel mTORC1/2 dual inhibitor, against renal cell carcinoma. Cancer Lett..

[8] Korets SB, Musa F, Curtin J, Blank SV, Schneider RJ (2014). Dual mTORC1/2 inhibition in a preclinical xenograft tumor model of endometrial cancer. Gynecol. Oncol..

[9] Hassan B (2014). Catalytic mTOR inhibitors can overcome intrinsic and acquired resistance to allosteric mTOR inhibitors. Oncotarget.

[10] Voss MH (2020). Phase 1 study of mTORC1/2 inhibitor sapanisertib (TAK-228) in advanced solid tumours, with an expansion phase in renal, endometrial or bladder cancer. Br. J. Cancer.

[11] Mondesire WH (2004). Targeting mammalian target of rapamycin synergistically enhances chemotherapy-induced cytotoxicity in breast cancer cells. Clin. Cancer Res..

[12] Kannan K (2013). Abstract B189: MLN0128, an investigational mTORC1/2 inhibitor, demonstrates potent antitumor activity alone and in combination with paclitaxel in preclinical models of endometrial cancer. Mol. Cancer Therapeutics.

[13] David-West G, Ernlund A, Gadi A, Schneider RJ (2018). mTORC1/2 inhibition re-sensitizes platinum-resistant ovarian cancer by disrupting selective translation of DNA damage and survival mRNAs. Oncotarget.

[14] Shimizu T (2022). A phase 1 study of Sapanisertib (TAK-228) in East Asian patients with advanced nonhematological malignancies. Target Oncol..

[15] Fraenkel M (2008). mTOR Inhibition by Rapamycin Prevents β-Cell Adaptation to Hyperglycemia and Exacerbates the Metabolic State in Type 2 Diabetes. Diabetes.

[16] Lessnick SL, Ladanyi M (2012). Molecular pathogenesis of Ewing sarcoma: new therapeutic and transcriptional targets. Annu. Rev. Pathol..

[17] Fogarty NME (2017). Genome editing reveals a role for OCT4 in human embryogenesis. Nature.

[18] He D, Zhang X, Tu J (2020). Diagnostic significance and carcinogenic mechanism of pan-cancer gene POU5F1 in liver hepatocellular carcinoma. Cancer Med..

[19] Subbiah V (2013). Morphoproteomic profiling of the mammalian target of rapamycin (mTOR) signaling pathway in desmoplastic small round cell tumor (EWS/WT1), Ewing’s sarcoma (EWS/FLI1) and Wilms’ tumor(WT1). PLoS ONE.

[20] Huang HJ (2011). R1507, an anti-insulin-like growth factor-1 receptor (IGF-1R) antibody, and EWS/FLI-1 siRNA in Ewing’s sarcoma: convergence at the IGF/IGFR/Akt axis. PLoS ONE.

[21] Pavarana M (2004). Platinum and etoposide as second-line chemotherapy in advanced adult soft tissue sarcomas. J. Clin. Oncol..

[22] Neshat MS (2001). Enhanced sensitivity of PTEN-deficient tumors to inhibition of FRAP/mTOR. Proc. Natl Acad. Sci. USA.

[23] Guertin DA (2009). mTOR complex 2 is required for the development of prostate cancer induced by Pten loss in mice. Cancer Cell.

[24] Mao N (2021). Defining the therapeutic selective dependencies for distinct subtypes of PI3K pathway-altered prostate cancers. Nat. Commun..

[25] Eisenhauer EA (2009). New response evaluation criteria in solid tumours: revised RECIST guideline (version 1.1). Eur. J. Cancer.

